# *Rhizopus microsporus* Infections Associated with Surgical Procedures, Argentina, 2006–2014 

**DOI:** 10.3201/eid2605.191045

**Published:** 2020-05

**Authors:** Jolene R. Bowers, Juan Monroy-Nieto, Lalitha Gade, Jason Travis, Nicolás Refojo, Ruben Abrantes, Jorge Santander, Chris French, María Cecilia Dignani, Alejandra Ines Hevia, Chandler C. Roe, Darrin Lemmer, Shawn R. Lockhart, Tom Chiller, Anastasia P. Litvintseva, Liliana Clara, David M. Engelthaler

**Affiliations:** Translational Genomics Research Institute, Flagstaff, Arizona, USA (J.R. Bowers, J. Monroy-Nieto, J. Travis, C. French, D. Lemmer, D.M. Engelthaler);; Centers for Disease Control and Prevention, Atlanta, Georgia, USA (L. Gade, S.R. Lockhart, T. Chiller, A.P. Litvintseva);; Departamento Micología, Instituto Nacional de Enfermedades Infecciosas “Dr. Carlos G. Malbrán,” Buenos Aires, Argentina (N. Refojo, R. Abrantes, A.I. Hevia);; Asociación Argentina de Artroscopía, Buenos Aires (J. Santander, M.C. Dignani);; Pathogen and Microbiome Institute, Flagstaff (C.C. Roe);; Infection Committee of the Italian Hospital of Buenos Aires, Buenos Aires (L. Clara)

**Keywords:** *Rhizopus microsporus*, outbreak, whole-genome sequencing, surgical infections, surgical procedures, fungi, Argentina

## Abstract

*Rhizopus* spp. fungi are ubiquitous in the environment and a rare but substantial cause of infection in immunosuppressed persons and surgery patients. During 2005–2017, an abnormally high number of *Rhizopus* infections in surgery patients, with no apparent epidemiologic links, were reported in Argentina. To determine the likelihood of a common source of the cluster, we performed whole-genome sequencing on samples collected during 2006–2014. Most isolates were separated by >60 single-nucleotide polymorphisms, and we found no evidence for recombination or nonneutral mutation accumulation; these findings do not support common source or patient-to-patient transmission. Assembled genomes of most isolates were ≈25 Mbp, and multiple isolates had substantially larger assembled genomes (43–51 Mbp), indicative of infections with strain types that underwent genome expansion. Whole-genome sequencing has become an essential tool for studying epidemiology of fungal infections. Less discriminatory techniques may miss true relationships, possibly resulting in inappropriate attribution of point source.

Mucormycosis is a debilitating fungal infection; the mortality rate among persons with predisposing factors such as skin trauma (e.g., surgery), diabetes mellitus, or organ transplant is high. The fungus can be directly inoculated into a wound or inhaled (*1*–*3*). *Rhizopus* spp. are the Mucorales fungi that most commonly cause mucormycosis (*1*,*2*,*4*) and are the most common non-*Aspergillus* cause of invasive filamentous fungal infections (*5*). However, although Mucorales fungi are ubiquitous in the environment, mucormycosis is relatively uncommon. 

*Rhizopus microsporus* has been shown to be a cause of serious infections after anterior cruciate ligament reconstruction surgeries in Argentina (*6*,*7*). A recent review of 40 *Rhizopus*-associated cases of osteomyelitis that developed after these surgeries from 2005 through 2017 in several regions across Argentina identified 3 species—*R. microsporus* var. *rhizopodiformis, R. microsporus* var. *microsporus,* and *R. arrhizus* [syn. *R. oryzae*]—and implicated healthcare practices and facility shortcomings in the infections (*8*). Limited molecular analyses of osteomyelitis-associated *R. microsporus* infections identified commonalities among isolated strains (*7*); however, no genomic epidemiologic analyses have been performed on this nosocomial cluster. In this study, we analyzed the genomes of *R. microsporus* var. *rhizopodiformis* isolates from patients from multiple facilities in Argentina in the context of unrelated controls from outside the geographic area to empirically establish the relationships among them and determine whether infections may have originated from a common source.

## Materials and Methods

During 2006–2014, we collected 24 *R. microsporus* isolates from patients at 14 healthcare facilities in 10 provinces in Argentina (*8*). For unrelated control isolates, used to establish genomic context for the nosocomial cluster in Argentina, we selected 13 isolates from the US Centers for Disease Control and Prevention (Atlanta, GA, USA), collected from 2003 through 2015 (Table).

**Table:** Characteristics for controls and patients with *Rhizopus microsporus* infection associated with surgical procedures in Argentina, 2006–2014*

Isolate	*R. microsporus* variety	Genome assembly size, Mbp	GC content, %	Year	Surgery	Isolate site	Patient age, y	US state/Argentina province	
Control†									
B05459	*Rhizopodiformis*	25.1	37.2	NA	NA	NA	NA	NA	
B06590	*Oligosporus*	45.3	37.1	2003	NA	NA	NA	NA	
B06600	*Oligosporus*	46.5	36.6	2003	NA	NA	NA	NA	
B07367	*Rhizopodiformis*	25.0	37.3	2008	NA	NA	NA	Georgia	
B07386	*Microsporus*	30.2‡	39.1‡	2008	NA	Skin	NA	NA	
B07585	*Microsporus*	29.2‡	39.5‡	2009	NA	NA	NA	NA	
B07643	*Rhizopodiformis*	25.1	37.3	2009	NA	NA	NA	Georgia	
B07675	*Rhizopodiformis*	25.1	37.3	2009	NA	Chest tissue	NA	Utah	
B08956	*Microsporus*	28.7‡	40.3‡	2010	NA	Respiratory	NA	Georgia	
B10187	*Rhizopodiformis*	25.1	37.3	2013	NA	Wound	NA	Georgia	
B10548	*Rhizopodiformis*	25.1	37.3	2013	NA	NA	NA	Pennsylvania	
B10881	*Rhizopodiformis*	25.1	37.3	2014	NA	Axilla tissue	NA	Colorado	
B11147	*Rhizopodiformis*	25.1	37.3	2015	NA	BAL	NA	Colorado	
Patient†									
B11523	*Rhizopodiformis*	25.5	37.3	2011	Other (chest)	Sternum muscle	<18	Caba	
B11526	*Rhizopodiformis*	25.1	37.3	2010	Knee	Bone, soft tissue biopsy	18–35	Mendoza	
B11529	*Rhizopodiformis*	25.1	37.3	2011	Knee	Knee	18–35	Entre Rios	
B11531	*Rhizopodiformis*	25.1	37.3	2006	Knee	Bone	18–35	Santa Fé	
B11532	*Rhizopodiformis*	25.1	37.3	2011	Renal transplant	Abdominal fluid	35–65	Tucuman	
B11533	*Rhizopodiformis*	27.7	37.3	2011	Renal transplant	Abdominal fluid	35–65	Tucuman	
B11534	*Rhizopodiformis*	25.1	37.3	2010	Knee	ACL	18–35	Mendoza	
B11535	*Rhizopodiformis*	25.1	37.3	2011	Unknown	Surgical site	<18	Caba	
B11538	*Rhizopodiformis*	25.1	37.3	2011	Other (chest)	Surgical site	<18	Caba	
B11539	*Rhizopodiformis*	25.1	37.3	2011	Renal transplant	Surgical site	35–65	Entre Rios	
B11540	*Rhizopodiformis*	25.1	37.3	2011	Renal transplant	Abdominal fluid	35–65	Tucuman	
B11541	*Microsporus*	50.1	37.3	2011	Renal transplant	Surgical site	35–65	Entre Rios	
B11543	*Rhizopodiformis*	25.1	37.3	2011	Knee	Femur	18–35	Entre Rios	
B11546	*Rhizopodiformis*	25.1	37.3	2011	Other (hip replacement)	Hip	>65	Corrientes	
B11547	*Rhizopodiformis*	25.1	37.3	2006	Knee	Femur	18–35	San Juan	
B11549	*Rhizopodiformis*	25.0	37.3	2011	Environmental surface	Environmental surface		Entre Rios	
B11550	*Rhizopodiformis*	25.0	37.3	2008	Knee	Knee	18–35	Gran Buenos Aires	
B11551	*Rhizopodiformis*	25.1	37.3	2010	Other (unknown)	Abdominal cavity	<18	Salta	
B11552	*Rhizopodiformis*	25.1	37.3	2010	Renal transplant	Surgical site	35–65	Entre Rios	
B11553	*Rhizopodiformis*	25.2	37.3	2011	Knee	Knee	18–35	Cordoba	
B11554	*Rhizopodiformis*	45.7	37.1	2014	Knee	Bone	18–35	Santa Fe	
B11555	*Rhizopodiformis*	25.5	37.3	2011	Other (chest)	Pericardial fluid	<18	Caba	
B11556	*Rhizopodiformis*	25.1	37.3	2009	Knee	Knee	<18	Gran Buenos Aires	
B11557	*Microsporus*	43.7	37.2	2013	Renal transplant	Renal tissue	35–65	Salta	

*ACL, anterior cruciate ligament; BAL, broncho-alveolar lavage sample; NA, not available. †Controls from the Centers for Disease Control and Prevention, 2003–2015; patients from Argentina with *R. microsporus* infection associated with surgical procedures, 2006–2014. ‡These genomes include sequence for *Burkholderia rhizoxinica*, a known symbiont of *Rhizopus*.

We extracted DNA from the 37 isolates by using a DNeasy Blood and Tissue Kit (QIAGEN, https://www.qiagen.com), according to the manufacturer’s recommendations. Genomic DNA was fragmented to ≈500 bp by using a QSonica Q800R2 Sonicator (https://www.sonicator.com), genome libraries were prepared for paired-end sequencing and quantified by using a KAPA Hyper Prep Kit and KAPA Library Quantification Kit (KAPA Biosystems, https://sequencing.roche.com), and 33 samples were sequenced on the Illumina NextSeq at 150 × 150–bp reads and 4 samples on the Illumina MiSeq at 300 × 300–bp reads (both https://www.illumina.com). We deposited Illumina read data in the National Center for Biotechnology Information Sequence read archive (https://www.ncbi.nlm.nih.gov/sra) under BioProject PRJNA526061. We also prepared sample B11533 for long-read sequencing. We extracted high molecular weight DNA with the MasterPure Yeast DNA Purification Kit (Lucigen Epicentre, https://www.lucigen.com) by using a nonenzymatic method for lysis targeting a 20-kb insert size. We performed single-molecule real-time (SMRT) sequencing by using the PacBio RS II SMRT DNA sequencing system (Pacific Biosciences, https://www.pacb.com) as previously described (*9*). Specifically, we generated 20-kb libraries with the SMRTbell template prep kit 1.0 (Pacific Biosciences). We bound libraries to polymerase by using a DNA/Polymerase Binding Kit P6 v2 (Pacific Biosciences), loaded on 2 SMRT cells (Pacific Biosciences), and sequenced with C4 v2 chemistry (Pacific Biosciences) for 360-min movies. We downloaded the public reference genomes of comparable size to the samples in this study—GCA_002083735 (ATCC 11559), GCA_002708625 (ATCC 52813), and GCA_002083745 (ATCC 52814)—and used them for comparison.

We assembled short read data by using UGAP (https://github.com/jasonsahl/UGAP), which uses the SPAdes genome assembler (*10*), and assembled the PacBio long reads of sample B11533 by using Canu (*11*); we performed error correction by using the Illumina short reads in 6 rounds of Pilon (*12*). Whole-genome single-nucleotide polymorphism (SNP) typing (WGST) included only the 32 genomes (of 37 total) that assembled to ≈25 Mbp. For WGST, we generated SNP matrices to identify point mutations among the isolates (and thus infer strain relatedness) with NASP (*13*), in which reads were aligned to the assembly of sample B11533 by using the Burrows-Wheeler Alignment tool (*14*). We called SNPs with the Genome Analysis Toolkit (*15*) and included them in further analyses only if they were present in all samples, covered by >10× depth with >90% consensus in each sample and not in any duplicated regions in the reference genome as identified by NUCmer (*16*). The resulting SNP matrix comprised the core genome common to all samples in the analysis. We performed maximum-likelihood phylogenetic analyses with IQ-TREE (*17*) and maximum-parsimony analyses with MEGA version 7.0 (*18*), and we constructed phylogenetic trees in iTOL version 3 (*19*).

We assessed the spatial distribution of SNPs among the *Rhizopus* genomes by using RecomboMamba, which is part of the RECAP toolbox (https://github.com/TGenNorth/RECAP). RecomboMamba was designed to easily detect regions of relatively high SNP density that may indicate recombination or regions under selection that may confound phylogenetic inference. It uses output from an SNP analysis pipeline and a sliding window to tally the numbers of SNPs for each sample by reference genome position to build a graphic display of SNP density, read depth, and pairwise homoplasy index (*20*).

## Results

Read lengths from the PacBio sequencing of B11533 averaged 2,175 bp. The assembly of PacBio and Illumina data of this genome resulted in a genome size of 27.7 Mbp. Approximately 22.6% of the genome was identified as repeat regions according to NUCmer (*16*) in the NASP analysis, which is consistent with the size and repeat region variation characteristic of *Rhizopus* (*4*). We uploaded this assembly into GenBank (accession no. SMRR00000000).

### Genomic Relationships among Isolates

Using the whole-gene sequencing (WGS) data**,** we confirmed that most (22 of 24) of the isolates from the Argentina cluster were *R. microsporus* var. *rhizopodiformis* by WGST and by 18S, internal transcribed spacer, 28S, and *act1* genetic typing (*21**,**22*). A total of 21 isolates fell into a single clade that also included 3 controls and the publicly available genome of the American Type Culture Collection (ATCC) 11559 strain, first described in 1935 in the USSR (*23*), with 3,170 SNPs among them (Figure). The isolates were collected from patients who had undergone various types of surgeries, encompassing a wide geographic range across multiple years (Figure), and from patients of various ages (Table). Most of the Argentina cluster isolates (n = 17) formed a well-supported inner clade consisting of 1,235 SNPs (Figure). Although 2 sets of epidemiologically related isolates were separated by <20 SNPs, the closest relationship between any other 2 isolates in the tree was 60 SNPs (range 60–912, mean 430), a considerable evolutionary distance, not indicative of a recent transference. A set of 3 samples from the same patient (B11523, B11538, and B11555) were appropriately closely related; the first 2 isolates were identical (i.e., 0 SNPs) and the third was separated by 17 SNPs. One pair of isolates outside the large cluster clade (B11529 and B11543) were separated by 6 SNPs and were collected from 2 patients from the same facility, whose surgeries were 3 weeks apart. This low number of SNPs is characteristic of recent direct transmission or indirect transmission from a common source. The SNP-based phylogenetic analysis included 21.3 Mbp, which covers 99% of the 21.5 Mbp of the unduplicated reference genome of B11533, derived from the 27.7-Mbp full assembly minus the 22.6% of the genome identified as repeat regions. The 21.3-Mbp finding indicates that insertions/deletions may have played a small, if any, role in the evolutionary history of this sample set.

**Figure:**
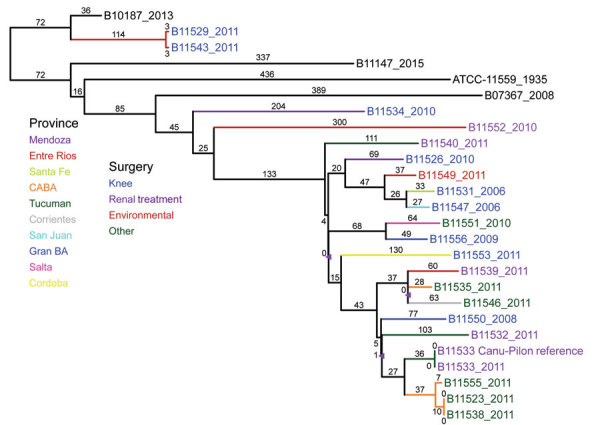
Maximum parsimony phylogenetic analysis, including 100 bootstrap statistical replicates, and epidemiologic data for *Rhizopus microsporus* var. *rhizopodiformis*, including 21 isolates from Argentina, 2006–2014; 3 control isolates (in black); and the public genome of American Type Culture Collection 11559, highlighting a lack of solid evidence for a common source for the surgical infections. Branch colors denote the province; isolate name colors denote the surgery type. The single-nucleotide polymorphism–based comparison covered 21.3 Mbp (99% of the 21.5 Mbp of the unduplicated reference genome of B11533). The consistency index is 0.99 out of 1.00, indicating a low level of potential homoplasy in the dataset. The 3 tree nodes with <90% bootstrap statistical support are marked with a triangle.

We found no apparent evidence of recombination or selective force in the SNP distribution that could potentially skew phylogenetic inference across the 25 genomes (Appendix Figure 1). The even distribution of SNPs is characteristic of neutral point mutations.

### Genome Variability

Five isolates had assembled genome sizes of 43 to 51 Mbp; all other assemblies were ≈25 Mbp (Table). The 5 isolates included 3 from the Argentina cluster, of which 2 were typed as *R. microsporus* var. *microsporus* and 1 as *R. microsporus* var. *rhizopodiformis*, and 2 controls, which were typed as *R. microsporus* var. *oligosporus* by 18S, 28S, ITS, *act1*, EF-1α sequences (*21**,**22*). NASP analysis showed that these samples had multiple SNP states, indicating heterozygosity at several of the SNP loci identified by NASP. Heterozygosity may result from genome expansion and aneuploidy or sample mixtures. Including these 5 samples in the phylogenetic analyses made results inconclusive; therefore, we removed them from the analyses. Sequence data from 3 control samples (all *R. microsporus microsporus* isolated from skin, respiratory tract, and an unknown source) also contained sequences from *Burkholderia rhizoxinica* (Table), a known endosymbiont of some *R. microsporus* strains (*24*).

An analysis of all samples with ≈25-Mbp genomes (which included 11 control isolates, 3 genomes from public databases, and 21 Argentina cluster isolates) illustrated large genomic distances among different isolates of *R. microsporus*, even within a variety (Appendix Figure 2). SNP analysis identified 1.2 million SNPs, compared with 3,170 SNPs identified within the cluster. The phylogeny shows that the public genomes for ATCC 52813 and ATCC 52814 differ from the closest control isolates by >800k SNPs, despite all having been identified as *R. microsporus* var. *microsporus*, which is remarkable considering that *R. microsporus* var. *rhizopodiformis* differs from *R. microsporus* var. *microsporus* control isolates by >500k SNPs. Overall, the *R. microsporus* var. *rhizopodiformis* group is a relatively tight genomic cluster compared with *R. microsporus* var. *microsporus*, possibly because of sampling bias or differential rates of evolution (Appendix Figure 2).

## Discussion

The genomic diversity among isolates from the Argentina cluster of *R. microsporus* infections is not consistent with a point-source outbreak (*25*–*27*). We identified no associations between isolate phylogenetic placement and patient metadata, which included facility, procedure type, and province. Given the extent of genomic differences among the isolates from the cluster and the lack of associations between genotypes and epidemiologic factors, we found no evidence to support the hypotheses of patient-to-patient transmission or a common source. However, our data do not rule out the possibility of a common source because different strains or even species may come from a common source (*28*). In our case series, the most likely source of infection was environmental contamination at the facilities or during hospital practices (*8*); contamination of the operating room with unfiltered ambient air might be the common source. During our previous epidemiologic investigation (*8*), the only common factor among the patients studied was the use of an operating room (for arthroscopy of the knee for anterior cruciate ligament repair, placement of an implantable central venous catheter, or organ transplantation). This speculation is supported by findings from our previous epidemiologic investigation: operating rooms used for case-patients had no HEPA filters; if used, HEPA filters were not used properly; or the operating room was contaminated with unfiltered external air (*8*).

WGST has become an essential tool for investigating outbreaks of fungal infections; however, defining levels of SNP identity among isolates to determine relatedness remains challenging. Recent WGST analyses of several fungal infection outbreaks help shape our understanding about the relatedness of isolates from point-source outbreaks (*25*–*27*,*29*,*30*). However, WGS data from clusters not linked to a common source are scarce, and information about the expected genomic diversity among strains from the same region that cannot be linked to a common source is lacking. On the basis of WGS from outbreaks with strong epidemiologic data implicating a common source, isolates that differ by <10 SNPs are considered to be nearly identical and to originate from the same source; isolates sharing tens or hundreds of SNPs are considered different. However, these thresholds are arbitrary, dependent on bioinformatics pipelines, and species specific. To address this issue, Chow et al. defined pairwise SNP distances among isolates of *Candida auris* from the same patient as an identity reference point (*31*). Specifically, outbreak isolates are considered to be of the same origin if the number of SNPs between them is the same or lower than the average number of SNPs between multiple isolates from the same patient or known source. Although developed specifically to determine transmission of *C. auris*, this approach can be adapted to other species and outbreak situations if multiple isolates from the same patients are collected. The number of SNPs separating genomes in the inner clade of the phylogeny generated in this study, which included most of the Argentina isolates from the cluster, was relatively low compared with the number of SNPs separating genomes of the control isolates. However, this number was higher than the differences among multiple isolates from a single patient. Specifically, 0–17 SNPs separated isolates from the same patient, and 60–762 SNPs separated strains from different patients and different facilities. One case of apparent nosocomial transmission was identified in which 2 isolates from 2 patients admitted to the same hospital within 3 weeks differed by 6 SNPs. Because *Rhizopus* spp. infection is not contagious, transmission probably occurred through the contaminated equipment or from the same environmental source.

Because fungal genomes are large and highly complex, thousands of SNPs separating conspecifics is not uncommon (*25*,*26*,*29*), which is illustrated here within the *R. microsporus* var. *microsporus* group. The relatively low numbers of SNPs separating genomes in the inner clade is consistent with a common geographic origin and suggest a relatively recent common ancestor for these 17 isolates. Such limited population diversity is similar to that found for recently emerged fungal populations that display years to decades of evolution in a restricted geographic locale, such as the emergent clones of *Cryptococcus gattii* in the Pacific Northwest (*32*) and the recently described clonal population of *Coccidioides immitis* in southeastern Washington state (*33*).

Although the mutation rate within *R. microsporus* is not known, we found no association between genetic distance and sampling dates by using root-to-tip regression analysis, which suggests a lack of molecular clock–like behavior. Furthermore, we found no apparent evidence of recombination or mutation selection in the even SNP distribution across the 25 genomes, suggesting that most SNPs resulted from neutral point mutations and showing that these samples are separated by substantial amounts of evolution, which is not typical of patient-to-patient or point-source outbreaks. However, we cannot rule out the possibility of rapid mutations occurring within a common-source outbreak or a well-established but minimally diverse common-source population. The recent global expansion of *C. auris* has advanced our knowledge of the varying evolutionary rate of nosocomial fungi; a recent analysis established a within-hospital rate of 5.7 × 10^–5^ nt substitutions/site/year (*34*). However, whether the mutation rate of *C. auris* is applicable to that of *Rhizopus* spp., a different taxonomic group with different ecology, remains unclear. Such a rate in *R. microsporus* would predict >1,425 SNPs between genomes separated by only a year. The inclusion of the ATCC 11559 control strain, isolated in 1935 in the Soviet Union, indicates that hypermutation is not occurring because this 84-year-old strain is separated from the Argentina cluster clade by <800 SNPs.

*Rhizopus* spp. are known to undergo chromosomal duplication events and potentially cross-species hybridization and to contain large proportions of inactive transposable elements (*4*), which may explain the vast differences in genome sizes and multiple SNP states (i.e., heterozygosity) detected at many genomic loci in the samples with 43–51 Mbp assembled genome sizes. The public genomes for *R. microsporus* in GenBank are also of various assembly sizes, ranging from 24.1 Mbp (GenBank accession no. GCA_002083735) to 75.1 Mbp (GCA_000697275). A substantial expanse of genome size variation could also result from suboptimal sequence data quality or read length, preventing proper contig formation during assembly and overestimation of genome size or pileup of repeat regions, thereby leading to underestimation of genome size (*4*). However, our data were of high quality and SNPs were filtered for high-confidence SNPs, although strain mixtures cannot be ruled out. In addition, hybridization between *Rhizopus* species or subspecies varieties has been described (*4*), which would confound phylogenetic analysis. Last, many fungi carry bacterial endosymbionts that alter the assembled genome sizes and GC content, including some strains of *R. microsporus* and *Burkholderia rhizoxinicus* (*35*); *B. rhizoxinicus* often requires obligate symbiosis with *R*. *microsporus* (*36*) and provides a toxin for plant pathogenesis to its host (*36**,**37*). In our sample set, 3 control isolates of *R. microsporus* var. *microsporus* from skin, respiratory tract, and an unknown source harbored *B. rhizoxinicus*. To our knowledge, whether the toxin or another factor from the symbiosis contributes to human infection has not been studied. Because many fungi are capable of these and other forms of genomic and chromosomal plasticity, phylogenetic analyses of fungal clusters, even in outbreak scenarios, need to account for these potentially confounding factors.

The cryptic diversity seen in this study might be missed by use of less discriminatory typing techniques, such as matrix-assisted laser desorption/ionization time-of-flight mass spectrometry or repetitive element palindromic PCR (*7*), possibly resulting in inappropriate point-source attribution. WGST has become the standard for molecular/genomic epidemiology, even (or especially) with understudied or rare pathogen events. However, despite the successful use of WGST to solve numerous medical and public health mysteries, the complexities of certain microbes and their resultant patient clusters are not always clarified, and without intensive sampling and routine genomic surveillance, causes of such clusters may remain hidden.

Appendix Additional results from study of *Rhizopus microsporus* infections associated with surgical procedures, Argentina, 2006–2014.
