# Supplementary material for: Rhizopus microsporus Infections Associated with Surgical Procedures, Argentina, 2006–2014
Source: Emerg Infect Dis. 2020 May;26(5):937–44. doi: 10.3201/eid2605.191045 (PMC7181922; doi:10.3201/eid2605.191045)
Supplement: Appendix — Additional results from study of Rhizopus microsporus infections associated with surgical procedures, Argentina, 2006–2014. [file 19-1045-Techapp-s1.pdf]

Article DOI: https://doi.org/10.3201/eid2605.191045

Rhizopus microsporus Infections Associated with Surgical Procedures, Argentina, 2006–2014

Appendix

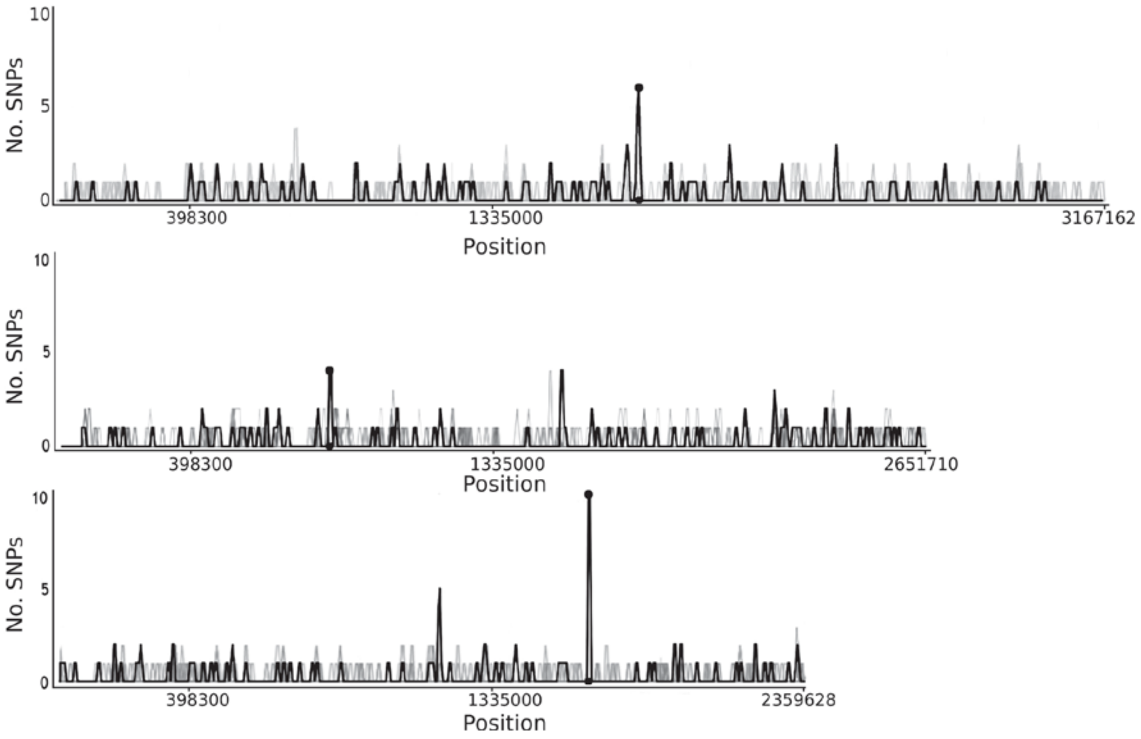

Appendix Figure 1. Output from RecomboMamba showing the even SNP distribution among all the genomes included in the phylogenetic analysis illustrated in Figure 1, across the largest three contigs of the reference genome, B11533. In this snapshot, the public genome of ATCC 11559 is the darkest line that shows a higher number of SNPs than other samples, reflecting the higher number of autologous SNPs in this genome compared to the reference.

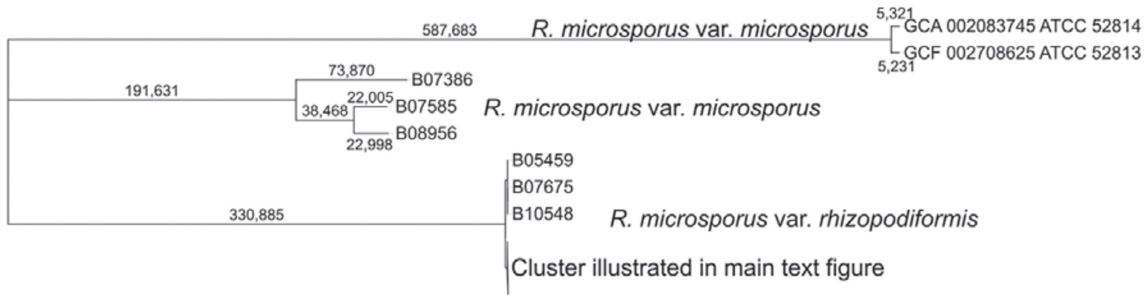

Appendix Figure 2. Maximum parsimony phylogenetic SNP analysis of 35 *R. microsporus* genomes, including 21 Argentinian isolates, 11 control isolates, and three public genomes, showing the genomic distances within and between *R. microsporus* varieties. The SNP-based comparison covered 17.8 Mbp (64.0% of the 27.7 Mbp reference genome of B11533). The consistency index is 0.98. Bootstrap values were >90% for all nodes.
